# Association Between Cortical Superficial Siderosis and Dementia in Patients With Cognitive Impairment: A Meta-Analysis

**DOI:** 10.3389/fneur.2019.00008

**Published:** 2019-01-29

**Authors:** Chenheng Zhou, Keqin Liu, Shenqiang Yan, Ying Jin

**Affiliations:** ^1^Department of Neurology, First People's Hospital of Wenling, Wenling, China; ^2^Department of Neurology, Hangzhou First People's Hospital, Hangzhou, China; ^3^Department of Neurology, The Second Affiliated Hospital of Zhejiang University, School of Medicine, Hangzhou, China; ^4^Department of Integrated Traditional Chinese and Western Medicine, First People's Hospital of Wenling, Wenling, China

**Keywords:** dementia, Alzheimer's disease, superficial siderosis, cognitive impairment, meta-analysis

## Abstract

**Background:** It remains unclear whether cortical superficial siderosis (cSS) is associated with dementia and its subtypes. We thus performed a meta-analysis to evaluate the relationship between dementia and cSS.

**Methods:** We searched EMBASE, PubMed, and Web of Science for relevant studies assessing risk of dementia and prevalence of cSS in patients with cognitive impairment. Fixed-effects and random-effects models were performed.

**Results:** Seven eligible studies including 3,218 patients with definite cognitive impairment were pooled in meta-analysis. The prevalence of cSS was 3.4%. The pooled analysis demonstrates odds ratio for cSS and dementia to be 1.60 (95% CI 1.04–2.44; *p* = 0.031). Subgroup analysis further indicated a significant association between cSS and Alzheimer's disease (AD) (OR = 2.01, 95% CI 1.34–3.02; *p* < 0.001), but not non-AD dementia (OR = 0.700, 95% CI 0.435–1.128; *p* = 0.143).

**Conclusions:** Our meta-analysis of available published data demonstrates an increased prevalence of dementia in the subjects with pre-existing cSS, especially for AD. These findings suggest cSS to be a candidate imaging indicator for AD. Further longitudinal research is needed to investigate the clinical relevance.

## Introduction

Dementia is a major public health concern associated with the aging population, and currently affects millions of individuals worldwide, while Alzheimer's disease (AD) is the most common cause of dementia in the elderly (1). The pathogenesis of AD consists of two parts, of which one is the amyloid cascade hypothesis, linked to cerebral amyloid angiopathy (CAA), and the other is the vascular hypothesis, linked to cerebral small vessel disease (CSVD) (2).

Cortical superficial siderosis (cSS) is characterized by linear hypointensities over the cortical surface of the supratentorial cerebral convexities on gradient recalled echo (GRE) or susceptibility weighted imaging (SWI) (3). The underlying pathological mechanism of cSS remains elusive, and generally assumed to reflect recurrent blood leaking episodes in the subarachnoid space (4). Patients with probable CAA manifested a much higher prevalence of cSS (34%) (5), compared with those from the general population (0.7%) (6). Moreover, Shams et al.'s study showed a link between cSS and the neuroimaging markers of CSVD (7). We therefore hypothesized that cSS itself might be a significant predictor for dementia, especially AD.

However, few studies investigated the relationship between cSS and dementia, and the results are controversial. Zonneveld et al. found a higher prevalence of cSS in patients with AD than those with mild cognitive impairment (MCI) (8), whereas a recent study failed to demonstrate the diagnostic significance of cSS for AD (9). We thus performed a meta-analysis to determine whether associations between cSS and dementia or AD exist in patients with cognitive impairment.

## Materials and Methods

We adhered to the Preferred Reporting Items for Systematic Reviews and Meta-Analyses (PRISMA) and Meta-analysis of Observational Studies in Epidemiology (MOOSE) statement (10, 11).

### Search Strategy and Eligibility Criteria

We searched appropriate articles by systematic queries of NCBI (PubMed), ISI Web of Science, and EMBASE databases on the 10th of September 2018, using the following search terms: “superficial siderosis” in association with “dementia” or “Alzheimer” or “cognition” or “cognitive.” Articles not published in English were translated and case reports were excluded. The references of all identified publications were reviewed for any additional studies not indexed. Two authors identified potentially relevant studies, resolving any uncertainties with a third author.

Both retrospective and prospective studies were eligible for inclusion if they (1) assessed the cognitive status for each subject in the cohort, and (2) provided the detailed data of cSS in each group according to the cognitive status.

### Study Selection and Data Extraction

Two authors considered all titles and abstracts for eligibility in a systematic manner, went through all articles selected as relevant and extracted data independently. We extracted information on study design, MRI parameters for cSS detection, definition of cSS, criteria of neuropsychological assessment, number, and demographics of participants (including age and sex), mini mental state examination (MMSE) score of participants, number of participants with cSS, number of participants of different cognitive status, and the severity of cSS (focal or disseminated) by using a unified data form. Discrepancies were resolved by consensus.

### Data Analysis

We used a fixed effects model (Mantel and Haenszel method) to calculate the pooled ORs and corresponding 95% confidence intervals (CIs), with weights calculated using the inverse variance method, because of the relatively small number of the outcome events. Subgroup analysis was performed to isolate patients with AD only. Statistical heterogeneity was assessed using I-squared statistics with inspection of the forest plot. Publication bias was evaluated with Egger's test, Begg's test, and the funnel plot. We repeated all analyses using random-effects models. All statistical analysis was performed with Stata 11.2 (StataCorp LP, Texas, USA).

## Results

**Figure 1:**
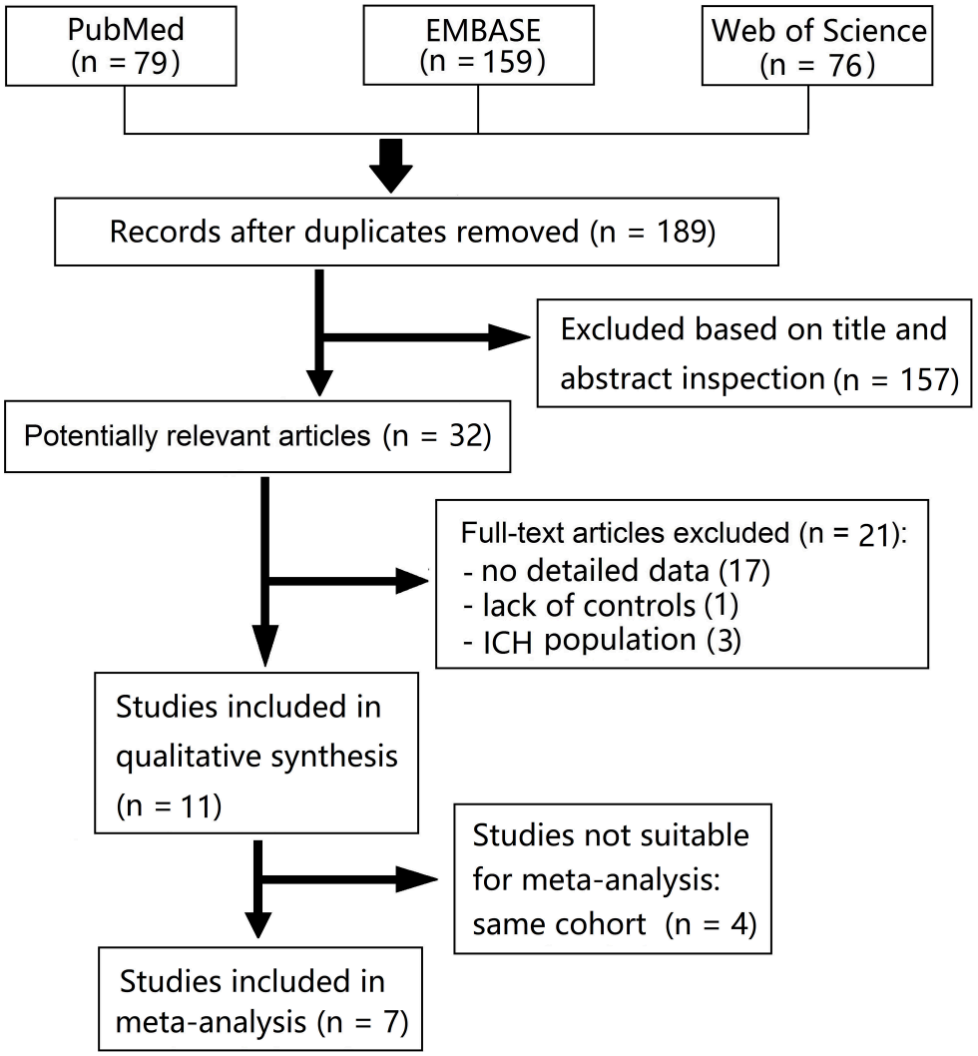
Flow diagram of literature search and study selection.

We identified 79 articles from PubMed, 159 from EMBASE, and 76 from Web of Science in our initial search. Fourteen studies (all published) met our predetermined criteria, however, five of these were from a same cohort, and other three were from cohorts of intracerebral hemorrhage (ICH) population. Finally, seven studies were pooled in a meta-analysis (Figure 1) (7–9, 12–15). Characteristics of the included studies are summarized in Table 1. The definition of cSS was almost the same across all included studies: hypointense linear structures within the subarachnoid space or in the superficial layers of the cerebral cortex on GRE or SWI.

**Table 1:** Characteristics of included studies.

**Study reference**	**Design**	**Inclusion criteria**	**MRI parameters**		**Neuropsychological assessment**
			**Sequence**	**Field strength**
Kantarci et al. 12; Western cohort	Prospective^*^, the ADNI study	(1) Aged 55 and above; (2) elderly controls; (3) MCI subjects; (4) AD patients; (5) with T2^*^ GRE images	T2^*^-GRE	3.0 T	Clinical dementia rating core
Wollenweber et al. 13; Western cohort	Prospective, memory clinic patients	(1) Aged 50 and above; (2) with standardized MRI	T2^*^-GRE	3.0 T	MCI: Petersen criteria; dementia: ICD-10 criteria
Zonneveld et al. 8; Western cohort	Prospective, the Amsterdam Dementia Cohort	(1) Underwent standardized dementia screening; (2) with SWI sequence	SWI	3.0 T	MCI: Petersen criteria; AD: NINCDS-ADRDA criteria; VaD: NINDS-AIREN criteria
Na et al. 14; Asian cohort	Prospective, from Samsung Medical Center	(1) Diagnosed with cognitive impairment; (2) with PiB-PET and standardized MRI	T2^*^-GRE	3.0 T	MCI: Petersen criteria; AD: NINCDS-ADRDA criteria; VaD: DSM-IV
Charidimou et al. 15; Western cohort	Prospective, from out-patient memory clinic	(1) Suspected cognitive impairment; (2) with standardized MRI	T2^*^-GRE	3.0 T	Clinical Dementia Rating core
Inoue et al. 9; Asian cohort	Prospective, from the dementia clinic	(1) Suspected cognitive impairment; (2) with standardized MRI	SWI	3.0 T	MCI: IWG-MCI criteria; AD: NINCDS-ADRDA criteria; VaD: NINDS-AIREN criteria
Shams et al. 7; Western cohort	Prospective, the Karolinska Imaging Dementia Study	(1) Underwent dementia investigation; (2) underwent MRI with hemosiderin sensitive sequences	SWI or T2^*^-GRE	3.0 or 1.5 T	ICD-10 criteria

cSS, cortical superficial siderosis; the ADNI study, the Alzheimer's Disease Neuroimaging Initiative study; MCI, mild cognitive impairment; AD, Alzheimer's disease; GRE, gradient-recalled echo; ICD, international classification of diseases; SWI, susceptibility-weighted imaging; NINCDS-ADRDA, National Institute of Neurological and Communicative Disorders and Stroke-Alzheimer's Disease and Related Disorders Association; NINDS-AIREN, National Institute of Neurological Disorders and Stroke-Association Internationale pour la Recherché et l'Enseignement en Neurosciences; PiB-PET, pittsburg compound B-positron emission tomography; DSM-IV, Diagnostic and Statistical Manual of Mental Disorders-Fourth Edition; IWG-MCI, International Working Group on Mild Cognitive Impairment.

* *ANDI is a longitudinal multicenter natural history study for AD, while the data of cSS and cognition was cross-sectional*.

Study demographics are summarized in Table 2. These studies were composed of 4,005 patients with cSS evaluation (study sample size range: 212–1,504), 110 (2.7%) of which had cSS on initial GRE or SWI, while 4.9% of AD patients had cSS. The severity of cSS was classified as focal (restricted to ≤3 sulci) or disseminated (≥4 sulci) in six studies (7–9, 13–15), and with detailed data in three (7, 8, 14). The cognitive statuses were classified as AD, vascular dementia, other dementia or undetermined, MCI, subjective cognitive complains, or cognitively normal (Table 2).

**Table 2:** Study demographics and outcomes.

**Study reference**	**Kantarci et al. (12)**	**Wollenweber et al. (13)**	**Zonneveld et al. (8)**	**Na et al. (14)**	**Charidimou et al. (15)**	**Inoue et al. (9)**	**Shams et al. (7)**	**Total**
Population size	562	212	809	232	339	347	1,504	4,005
Age, y	–	74 (mean)	66 (mean)	72 (mean)	73 (mean)	74 (mean)	63 (mean)	67 (mean)
Male	295 (52.5%)	89 (42.0%)	450 (55.6%)	97 (41.8%)	148 (43.7%)	130 (37.4%)	709 (47.1%)	1,918 (47.9%)
MMSE score	–	26 (median)	24 (mean)	22 (mean)	–	21 (mean)	25 (mean)	24 (mean)
**COGNITIVE STATUS**								
AD	40 (7.1%)	Any dementia	249 (30.8%)	62 (26.7%)	86 (25.4%)	162 (46.7%)	423 (28.1%)	26.9%
VaD	–	84 (39.6%)	12 (1.5%)	74 (31.9%)	18 (5.3%)	28 (8.1%)	54 (3.6%)	5.8%
Other dementia or undetermined	–		237 (29.3%)	–	42 (12.4%)	74 (21.3%)	224 (14.9%)	19.2%
MCI	351 (62.5%)	128 (60.4%)	143 (17.7%)	96 (41.4%)	162 (47.8%)	51 (14.7%)	418 (27.8%)	33.7%
SCC or CN	171 (30.4%)	–	168 (20.8%)	–	31 (9.1%)	32 (9.2%)	385 (25.6%)	22.1%
cSS prevalence	6 (1.1%)	13 (6.1%)	17 (2.1%)	12 (5.2%)	10 (2.9%)	12 (3.5%)	40 (2.7%)	2.7%
Focal cSS	–	7	11	6	7	7	33	–
Disseminated cSS	–	6	6	6	3	5	7	–
CMB prevalence	90 (16%)	25 (12%)	214 (29%)^*^	108 (47%)	74 (22%)^&^	160 (46%)	288 (19%)^&^	24%
cSS prevalence in AD	1 (2.5)	–	12 (4.8%)	3 (4.8%)	5 (5.8%)	8 (4.9%)	21 (5.0%)	4.9%

MMSE, mini mental state examination; AD, Alzheimer's disease; VaD, vascular dementia; MCI, mild cognitive impairment; SCC, subjective cognitive complains; CN, cognitively normal; cSS, cortical superficial siderosis; CMB, cerebral microbleed.

* 749 of 809 subjects had available data of CMB.

& *Only the number of lobar CMB was given*.

**Figure 2:**
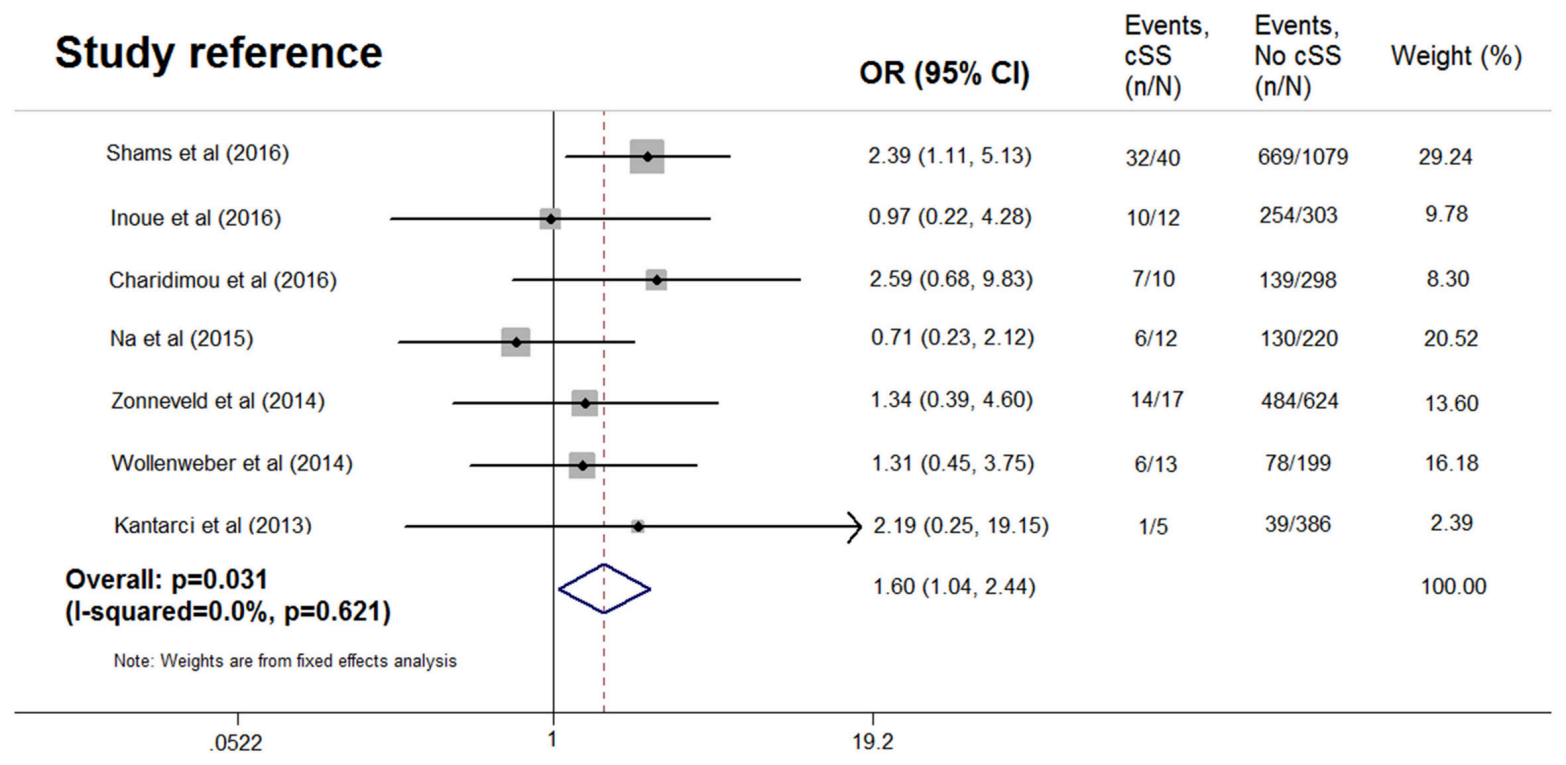
Meta-analysis of the association between dementia and cortical superficial siderosis in the subjects with cognitive impairment.

**Figure 3:**
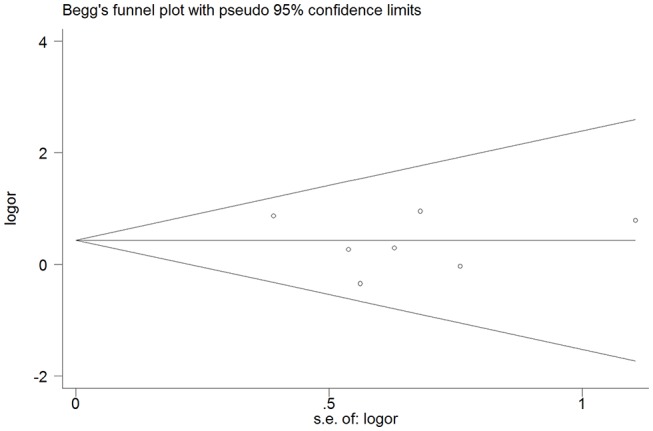
Publication bias from studies about the association between dementia and the presence of cortical superficial siderosis.

The cognitively normal patients (*n* = 171) and those with subjective cognitive complains (*n* = 616) were excluded, thus only patients with definite cognitive impairment were included in the meta-analysis. Among patients with dementia, 76 of 1,869 (4.1%) had cSS compared with 33 of 1,349 patients (2.4%) without dementia. Pooled analysis demonstrated OR for the presence of cSS and dementia to be 1.60 (95% CI 1.04–2.44; *p* = 0.031) with no evidence of statistical heterogeneity (*I*^2^ = 0.0%, *p* = 0.621) (Figure 2). There was no evidence of a publication bias either from the result of Egger's test (*p* = 0.604) or Begg's test (*p* = 0.881), and the shape of the funnel plot seemed symmetrical (Figure 3). After excluding the AD Neuroimaging Initiative study (12), which only enrolled AD patients, the association of cSS with dementia remained significant (OR = 1.156, 95% CI 1.028–1.301; *p* = 0.016).

**Figure 4:**
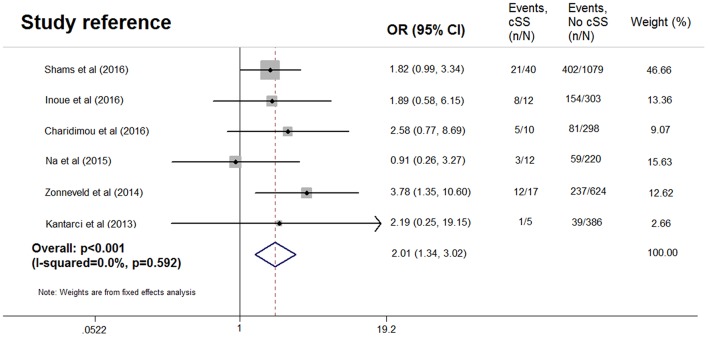
Meta-analysis of the association between Alzheimer's disease and cortical superficial siderosis in the subjects with cognitive impairment.

After excluding one study without data of dementia subtype (13), pooled analysis of the remaining six studies (7–9, 12, 14, 15), including 3,006 patients (96 with cSS), demonstrated OR for the presence of cSS and AD to be 2.01 (95% CI 1.34–3.02; *p* < 0.001) with no evidence of statistical heterogeneity (*I*^2^ = 0.0%, *p* = 0.592) (Figure 4), while no significant association was found between cSS and non-AD dementia (OR = 0.700, 95% CI 0.435–1.128; *p* = 0.143) (7–9, 14, 15). However, in the three studies with detailed data of cSS severity (7, 8, 14), the presence of disseminated cSS was not associated with dementia (OR = 0.873, 95% CI 0.337–2.260; *p* = 0.523), or AD (OR = 1.379, 95% CI 0.554–3.431; *p* = 0.976). All analyses were consistent when using a random-effects model.

## Discussion

Our meta-analysis in more than 3,000 subjects with cognitive impairment reported the existence of a positive relationship between cSS and AD, but not for non-AD dementia. These findings suggest cSS to be a candidate imaging indicator for AD.

Patients with cSS usually had lower cognitive scores (13). The Rotterdam Scan Study revealed a very low prevalence of cSS (0.7%) in a general population (6), while cSS was found in ~3% of patients in a memory clinic population, and with a prevalence of 5% in patients with AD (7–9, 13–15). Only one (0.001%, 1/787) subject with normal cognitive function was reported to have cSS in the studies considered (7–9, 12, 15). The abnormally low prevalence would reduce the efficacy of detecting significant effects, therefore, we only enrolled cognitive impairment patients in the current meta-analysis. Lummel et al. included in their study 113 subjects with non-traumatic and non-aneurysmal cSS, the most common etiologies was CAA, followed by reversible cerebral vasoconstriction syndrome, central nervous system vasculitis, and hyperperfusion syndrome (3). The clinical manifestations for cSS were: acute ICH: 49%; transient focal neurological episodes: 34%; cognitive impairment: 12%; generalized seizure: 4%; and headache: 2% (3).

The underlying mechanism of the pathological association between cSS and AD is not clear. The close relation between cSS and CAA might support the amyloid pathology. All of the individuals who presented with cSS in the Rotterdam Scan Study had cerebral microbleeds in lobar locations (6). The presence of cSS was also associated with lobar microbleeds in the memory clinic populations (7, 9, 15). In addition, the APOE genotype was more common in cases with cSS compared to those without (7, 14, 15). Immunohistochemistry staining showed severe CAA with A-β in the leptomeningeal and cortical vessels of a patient with both AD and cSS (16). Renard et al. evaluated cerebrospinal fluid amyloid-β 1–40 (Aβ40), amyloid-β 1–42 (Aβ42), total and phosphorylated-tau (t-tau and p-tau) in patients with symptomatic isolated cSS, and found that the patients with cSS showed higher t-tau and lower Aβ42 compared to the controls, and lower t-tau, p-tau, and Aβ40 compared to the AD patients (17). Moreover, *in vivo* amyloid imaging using [^11^C] Pittsburg compound B (PiB)-PET was performed in a cognitively impaired population, and cSS was found to be associated with higher global PiB retention ratio, and not present in any of the patients with a negative PiB scan (14), further supporting the hypothesis that cSS reflects an amyloid rather than the ischemic etiology.

Cognitive impairment was more frequent in patients with disseminated cSS, while transient focal neurological episodes were more often found in those with focal cSS (3). In the patients with spontaneous ICH, disseminated cSS was a key risk factor of new-onset dementia and recurrent symptomatic ICH (18, 19). However, the presence of disseminated cSS was not associated with dementia incidence in a recent longitudinal study of patients with probable CAA (OR = 1.268, 95% CI 0.702–2.292; *p* = 0.431) (20). Similarly, the severity of cSS could not predict dementia or AD in our meta-analysis. To sum up, the presence of disseminated cSS could predict dementia in patients with ICH, while it was not associated with dementia incidence in memory clinic populations. Disseminated cSS seems more important in the subjects with ICH, thus the characteristics of cohorts might be the key point. Considering the small sample size of patients with disseminated cSS, future studies are needed to investigate the clinical relevance of cSS severity.

Our study had several limitations. First, our analysis had inherent biases associated with the use of observational studies, and most of them were cross-sectional studies. All studies were subject to selection bias because not every individual underwent GRE or SWI. Moreover, the causality between cSS and dementia is still unclear, and future longitudinal studies are needed to clarify this association. Second, the use of unadjusted data rendered our analysis vulnerable to confounding variables, such as the neuroimaging markers of CSVD. Third, the clinical diagnostic criteria for dementia and its subtype might be not quite similar in each cohort.

In conclusion, our analysis shows that the presence of cSS is associated with AD. Future large multicenter studies and individual patient data meta-analyses are needed to investigate the importance of cSS in the pathogenesis and longitudinal progression of AD in the subjects with cognitive impairment.

## Author Contributions

YJ: design of the study, interpretation of data for the study, revision of the study for important intellectual content, and final approval of this version of the manuscript; CZ and KL: acquisition of data for the study, drafting of the study, revising the study for important intellectual content, and interpretation of data for the study; SY: acquisition of data for the study, drafting of the study, and revising the study for important intellectual content.

### Conflict of Interest Statement

The authors declare that the research was conducted in the absence of any commercial or financial relationships that could be construed as a potential conflict of interest.
